# *In silico* design and *in vivo* implementation of yeast gene Boolean gates

**DOI:** 10.1186/1754-1611-8-6

**Published:** 2014-02-02

**Authors:** Mario A Marchisio

**Affiliations:** 1 Department of Biosystems Science and Engineering (D-BSSE), ETH Zurich, Mattenstrasse 26, Basel 4058, Switzerland; 2 Current address: School of Life Science and Technology, Harbin Institute of Technology (HIT), 2 Yikuang Street, Nan Gang District, Harbin 150080, PR China

**Keywords:** Synthetic biology, Boolean gates, Yeast, Genomic integration, Computational design

## Abstract

In our previous computational work, we showed that gene digital circuits can be automatically designed in an electronic fashion. This demands, first, a conversion of the truth table into Boolean formulas with the Karnaugh map method and, then, the translation of the Boolean formulas into circuit schemes organized into layers of Boolean gates and Pools of signal carriers. In our framework, gene digital circuits that take up to three different input signals (chemicals) arise from the composition of three kinds of basic Boolean gates, namely YES, NOT, and AND. Here we present a library of YES, NOT, and AND gates realized via plasmidic DNA integration into the yeast genome. Boolean behavior is reproduced via the transcriptional control of a synthetic bipartite promoter that contains sequences of the yeast *VPH1* and minimal *CYC1* promoters together with operator binding sites for bacterial (i.e. orthogonal) repressor proteins. Moreover, model-driven considerations permitted us to pinpoint a strategy for re-designing gates when a better digital performance is required. Our library of well-characterized Boolean gates is the basis for the assembly of more complex gene digital circuits. As a proof of concepts, we engineered two 2-input OR gates, designed by our software, by combining YES and NOT gates present in our library.

## Background

A topic of growing interest in Synthetic Biology is represented by gene digital circuits. Primarily, they find application as biocomputing systems [1] and biosensors [2]. Their fundamental bricks, the Boolean gates, have been engineered into different chassis by exploiting various bio-chemical mechanisms. Transcriptional controls were shown to be able to reproduce, in bacteria, most of the two-input Boolean gates via just one or two regulated promoters [3]. A more complex design, including both promoter activation and tRNA-mediated translation regulation, was applied to the implementation of an AND gate in *E. coli*[4]. Boolean gates were engineered in yeast via mRNA structures such as ribozymes and riboswitches [5,6] whereas mammalian cells hosted more complex digital circuits based on RNA interference [7,8]. Translation regulation through mRNA-binding proteins was employed in mammalian cells for the construction of single-cell-based logic gates [9]. Finally, cell consortia were proved to be a solution for the modular design of logic circuits in yeast [10] and were applied to the building of a NOR gate in *E. coli*[11] too.

On the theoretical side we proposed a computational method for the automatic design of synthetic gene digital circuits [12]. Following a procedure borrowed from electronics, our algorithm employs the Karnaugh map method [13] to convert a truth table into the corresponding two Boolean formulas i.e. the Conjunctive (CNF) and the Disjunctive Normal Form (DNF) formulas. In electronics, CNF is called POS (Product Of Sums) since it is a logic multiplication (AND) of clauses (gates) that sum up (OR) the circuit inputs. In contrast, DNF performs a Sum Of Products (SOP) because it represents a logic addition of clauses where the circuit inputs are logically multiplied. Boolean formulas are translated into gene digital circuits organized in three layers of gates and *Pools* of signal carriers such as transcription factors, small RNAs, and chemicals [14].

Recently, we came up with a revised version of our computational tool where gene digital circuit design was simplified by adapting some ideas from [10] to the single cell scenario. In our framework, inputs for genetic circuits are chemicals that are divided into two classes depending on their action on transcription and translation [15]: *inducers* (transcription or translation activation) and *corepressors* (transcription or translation inhibition). Inducers bind either active repressors–turning them into an inactive configuration where they are no longer able to bind the DNA–or inactive activators that get activated and start recruiting RNA polymerases for mRNA synthesis. Corepressors bind inactive repressors–enabling them to bind promoters in competition with RNA polymerases–and active activators that become unable to get access to the DNA. At mRNA level, both inducers and corepressors bind riboswitches. Upon chemicals’ arrival to its aptamers, a riboswitch undergoes structural modifications that either produce (corepressor case) or remove (inducer case) a hairpin loop that prevents ribosome binding and translation initiation. Besides this direct control, translation is also negatively regulated by small antisense RNAs that base-pair to mRNA sequences (notice that each circuit scheme drawn by our tool is made of bacterial Standard Biological Parts from the MIT Registry– http://parts.igem.org–and it is therefore supposed to be hosted into prokaryotic cells).

These three mechanisms of transcription and translation control allow the construction of basic Boolean gates such as YES, NOT, and AND that are then assembled into digital circuits. YES gate do not have a counterpart in electronics and simply produce an output in presence of their (single) input. They are used to convert chemicals into transcription factors or small RNAs that carry out their action on other circuit’s gates. NOT gates return an output in absence of their input whereas AND gates take two inputs and produce an output only in presence of both inputs.

Circuits corresponding to SOP Boolean formulas assign a YES gate to each positive literal, a NOT gate to each negated one, and an AND gate to every clause. The OR gate that sums up the outputs of all the AND gates can be omitted by requiring that every AND gate produces the circuit output, a fluorescent protein. This is the so called *distributed output* architecture. POS formulas can be rearranged with the De Morgan’s laws [16] in such a way that they can also be mapped into circuits made of YES, NOT, and AND gates only. However, the common output of the AND gates is a transcription factor or an sRNA that regulates the only gate (NOT) responsible for fluorescence expression (*final gate* architecture). As a result, every digital circuit designed by our software is made of YES, NOT, and AND Boolean gates (for more details about our software, see [17] and Additional file 1: Figure S2).

In this work we present a library of YES, NOT, and AND gates engineered in yeast via *genomic integration* of bacterial (orthogonal) genes expressing repressor proteins (TetR, LacI, and LexA). Each construct owes its logic behavior to a *bipartite promoter*[18,19] regulated by one or two repressors that compete with RNA polymerases in order to get access to the DNA. The reproduction of bacterial regulation systems in yeast permitted us to follow both gate and circuit design supplied by our software. TetR and LacI are controlled by tetracycline and IPTG, respectively. LexA DBD (DNA-Binding Domain) was fused to the hormone-binding domain (HBD) of the human estrogen receptor such that it responds to *β*-estradiol [20] (Ottoz DSM, Rudolf F, Stelling J: unpublished). Although logic circuits have been already implemented in yeast [21], the ones we describe here are the *first* completely based on DNA *genomic integration*.

In the following, we give a careful description of the Boolean gates in our library and a detailed discussion of their performance. We point out how model-driven modifications–arising from considerations in [12] and better quantified with our new computational tool for eukaryotic gene circuit design [22] – permitted us to re-engineer two gates whose initial implementation did not work properly. We show how our basic gates can be composed into more complex circuits (OR gates) based on distributed output architecture. Finally, future improvements on gates’ design aimed at better performance and composability into larger networks are discussed.

## Results and discussion

### Gates’ construction: a bipartite promoter

**Figure 1:**
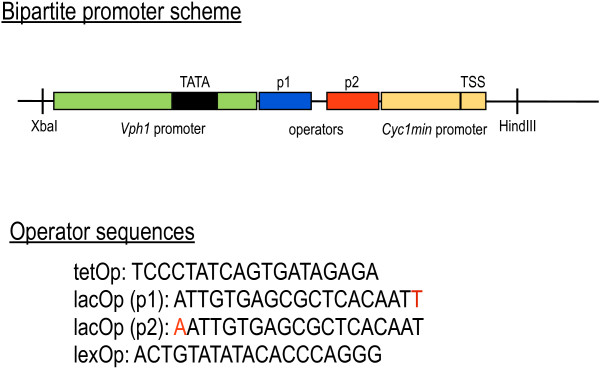
**A bipartite promoter.** Our bipartite promoter is made of fragments of the yeast *VPH1* and minimal *CYC1* promoters. The former provides the anchor point for RNA polymerases, the latter contains the TSS (see the Additional file 1 for their sequences). One or two operators are placed between the TATA box and the TSS such that repressor proteins can bind the DNA and inhibit transcription. TetOp, lacOp, and lexOp sequences are taken, in the order, from [32-34]. We modified the lacOp sequence with a T at the end–when it was placed close to the TATA box (position p1) or it was the only promoter operator–and with an A at the beginning, when it was inserted close to the TSS (position p2). In this way, the three operators used in this work had the same length (19 nucleotides).

Our basic Boolean gates exploit transcriptional repression. They are based on synthetic promoters where repressor operators are placed between the TATA box and the TSS (Transcription Start Site). In this way, it is possible to recreate in yeast the same competition, for the promoter sequence, that takes place in bacterial cells between RNA polymerases and repressors [23]. Once bound to the promoter, repressor proteins prevent RNA polymerase binding and inhibit transcription. Our synthetic promoters are bipartite [18]: they are made of a segment of the yeast *VPH1* promoter (containing the TATA box but excluding the two TSS [24]) and another small fragment of the yeast minimal *CYC1* promoter (where the TSS is well defined [25]). In between, we placed the repressor operators (see Figure 1). Each operator is 19 base-pair long and, when two operators are used, they are always separated by the same three nucleotides (CGT). Bacterial transcription factors have already been extensively used into eukaryotic cells. In literature one can find, for instance, a tetracycline-inducible promoter in *Schizosaccharomyces Pombe*[26]; a three-input logic gate in mammalian cells where the system tetracycline-tTA is employed [27]; a promoter regulated by LexA in *Saccharomices Cerevisiae*[28]; and a modified version of the yeast *ADH1* promoter able to bind LacI [29].

Each gate is realized into two configurations. YES and NOT gates are built with one and two operators. AND gates contain always two different operators: the two configurations come from the operators’ position swap with respect to the TATA box–where repression effects are stronger [30] – and the TSS. In analogy with our computational work, gates’ input are chemicals (tetracycline, IPTG, and *β*-estradiol) and the output is a protein (Citrine [31], which gives a yellow fluorescence signal).

Although gene Boolean gates are commonly characterized by the sole 1 to 0 output ratio (*ρ* = *V*_1*L*_/*V*_0*H*_, where *V*_1*L*_ is the minimal 1 output and *V*_0*H*_ is the maximal 0 output), we think that this parameter on its own cannot provide a comprehensive description of gates’ performance. Higher *ρ* values may simply mean a better repression rather than a clearer distinction between high and low output. Therefore, besides *ρ*, we calculated for each gate the *signal separation* (*σ* = *V*_1*L*_ - *V*_0*H*_) i.e. the distance, in arbitrary units (AU) of fluorescence, between the minimal 1 and the maximal 0 output at steady state [12] and what we called the *1-factor* (*φ* = *V*_1*c*_/*V*_1*o*_) that corresponds to the ratio of the 1 output of the *closed* gate– *V*_1*c*_ i.e. the gene expressing Citrine together with the gene(s) encoding for the repressor(s)–to the 1 output of the *open* gate (*V*_1*o*_–Citrine gene alone). In our work *φ* has a duplex meaning: one one hand it is an estimation of how effective inducer molecules (such as tetracycline and IPTG) are in inhibiting their target repressors, on the other hand it quantifies repression effects in absence of corepressors (as in the *β*-estradiol–LexA-HBD system).

### Single-input gates: YES and NOT

YES gates are devices that produce fluorescence in presence of a single input chemical. In our systems, a repressor protein (TetR or LacI) is expressed under the constitutive *ACT1* promoter. In absence of the corresponding input chemical (tetracycline or IPTG), the repressor binds a synthetic bipartite promoter at one or two operator sites and inhibits Citrine synthesis. Fluorescence is induced by the action of the input chemical that prevents its target repressor from DNA binding.

For our experiments we first determined, with a dose-response curve, the chemicals’ concentration necessary to fully deactivate the corresponding repressor in presence of a single operator on the target bipartite promoter. The measured quantities were considered as 1 input logic values for all the digital circuits we engineered. We found that 20*μM* tetracycline and 40 *mM* IPTG are required for a full transcription induction (see Additional file 1: Figure S4). In both cases, we did not detect any toxic effect on the cells.

**Figure 2:**
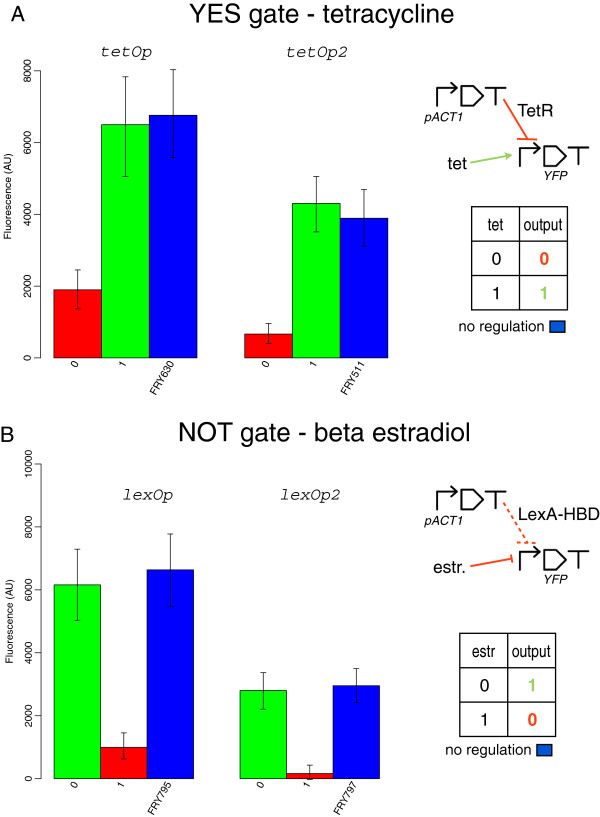
**Single-input gates.** **A)** Tetracycline responsive YES gates. **B)** *β*-estradiol sensing NOT gates. In both cases, a single operator guarantees higher signal separation whereas two operators permit to achieve a stronger repression. Fluorescence levels of the open gates (indicated with their yeast strain name–see Additional file 1) correspond to blue columns. Gates’ schemes are drawn with SBOL [36] icons (all the symbols used throughout the paper are shown in Additional file 1: Figure S1).

Both configurations of the tetracycline-based YES gate show a high signal separation (4601 AU with a single tet operator, 3640 AU with two tet operators–see Figure 2A) that permits to unequivocally distinguish between 0 and 1 output signals (no overlap of the error bars) and place the 0/1 threshold at 4000 AU (YES tetOp case) and 2500 AU (YES tetOp2). The single tet operator configuration, however, cannot achieve a strong repression. This causes a rather low 1 to 0 gain (*ρ* = 3.42) that is almost doubled in the tetOp2 design where, as expected [35], repression is much stronger. Finally, both variants have *φ* around 1. Therefore, we detected a high affinity between tetracycline and TetR and a moderate affinity between TetR and its operator binding site.

**Figure 3:**
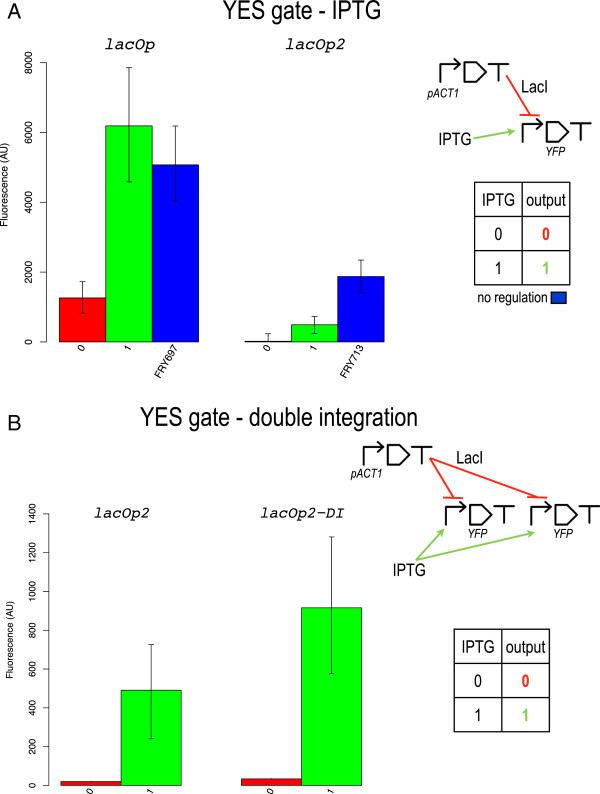
**IPTG responsive YES gates.** **A)** Single integration. **B)** lacOp2 double integration. The initial non-working YES gate based on two lac operators was rescued via a double integration of the transcription unit producing Citrine.

We found a similar trend in the IPTG-LacI system: a single lac operator (YES lacOp) gives a higher signal separation (4930 AU) whereas two lac operators permit to switch off fluorescence almost completely (see Figure 3A). However, the YES lacOp2 configuration presents a drawback: the bipartite promoter cannot be re-activated with 40 *mM* IPTG (higher concentrations were also of no use–data not shown). Indeed, the 1 output corresponds to only about one quarter of the open gate fluorescence and the signal separation (471 AU) is not high enough to determine a precise threshold between 0 and 1 fluorescent signals. According to our digital circuit computational analysis in [12], *σ* can be improved by increasing the transcription initiation rate of the promoter that leads to reporter protein production. Here, instead of re-engineering the lacOp2-containing bipartite promoter, we simply double integrated (DI) the transcription unit hosting it in order to mimic an increase of transcription initiation rate. As it is shown in Figure 3B, this procedure permitted to boost the 1 output (though still far from the YES lacOp2-DI open gate fluorescence that amounts to 4800.5 AU) without a significant increment in the 0 one. This allowed fixing an unequivocal 0/1 threshold at 500 AU.

Even though a single lac operator is not enough to drastically repress transcription and, as a consequence, its 1 to 0 ratio is slightly lower than 5, this configuration provides high values for both *σ* and *φ*. In contrast, the YES lacOp2-DI gate shows the best *ρ* (22.67) in our library. This underlines how this parameter alone cannot fully describe how well a genetic construct approximates a logic behavior. In this case, for instance, it hides a rather low signal separation (882 AU) and very low 1-factor (0.19 versus 1.22 in the single lac operator case). Below, we give a possible theoretical explanation of the different Boolean response with one and two lac operators. Moreover, we show how simulations of the YES lacOp2 and YES lacOp2-DI gates prove a correspondence between multiple integrations and higher transcription initiation rate.

NOT gates are made of two genes as well. LexA repressor is kept outside the nucleus by the HBD i.e. it is inactive in its ground configuration and gets activated only upon binding to *β*-estradiol. *β*-estradiol nullifies the HBD delocalizing action such that LexA can enter the nucleus and repress fluorescence. We chose 500 nM as 1 input level for *β*-estradiol. This concentration gives a stronger repression than the one registered both with TetR and LacI (see Additional file 1: Figure S4). Estradiol-responsive NOT gates do not differ, in performance, from the YES gates described above: higher signal separation for the single-operator configuration (NOT lexOp), better repression with two lex operators (NOT lexOp2–see Figure 2B). Both gates show fairly high values for *ρ* (more than 6 in the single and 17 in the double lexOp case) and *φ* slightly lower than 1 (this is probably due only to measure uncertainty rather than to LexA binding in absence of *β*-estradiol).

### Two-input gates: AND

We realized three kinds of two-input AND gates. Every AND gate contains two operators, each one binding a different repressor protein. As previously mentioned, AND gates have been engineered into two configurations by exchanging the positions of the two operators.

**Figure 4:**
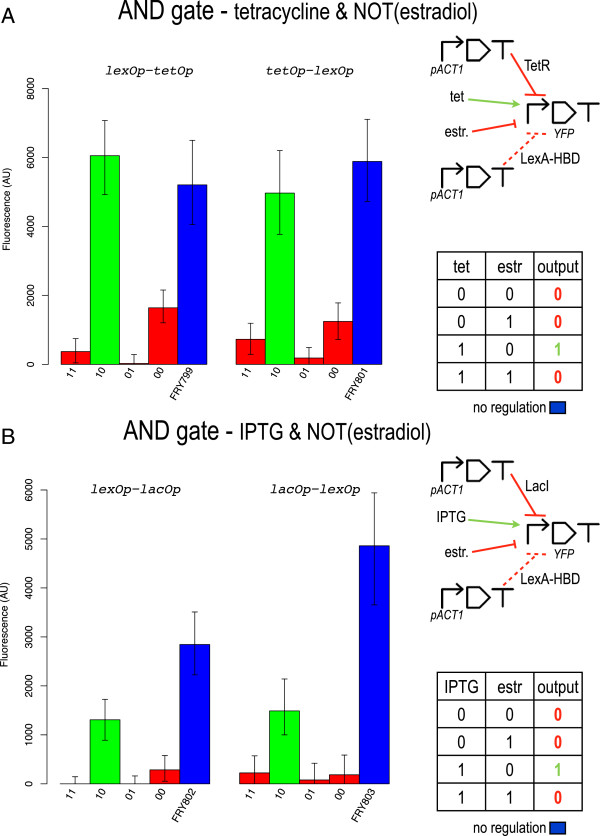
**AND gates (N-IMPLY) responsive to** ***β*-estradiol.** **A)** tet AND NOT(estr) gives the better performance in term of signal separation and 1-factor. **B)** IPTG AND NOT(estr) shows the best 1 to 0 gain among the AND gates in our library.

Overall, the best performance was obtained with the two "tet AND NOT(estr)" gates (to be precise, this Boolean gate is referred to as N-IMPLY in electronics). In particular, the design with the lex operator close to the TATA box (AND lexOp-tetOp) gives a better *σ* (see Figure 4A) whereas no significative difference between the two configurations is registered regarding *φ* and *ρ*. The AND lexOp-tetOp configuration, however, shows also that TetR is rather inefficient in repressing transcription when it binds in proximity of the TSS. In contrast, when the tet operator is placed close to the TATA box (AND tetOp-lexOp), TetR action is stronger and the difference among the three 0 output fluorescence levels is reduced.

**Figure 5:**
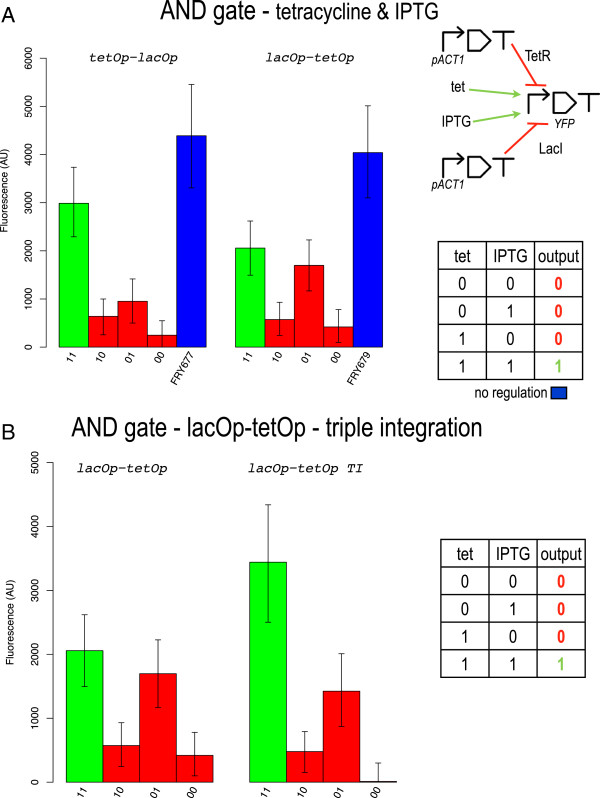
**AND gates responsive to tetracycline and IPTG.** **A)** Single integration. **B)** Triple integration (AND lacOp-tetOp configuration). Beside the expected increase in signal separation, the three 0 outputs are lower than in the single integration case. This is probably due to an accidental multiple integration of the LacI gene.

Tet operator positional effects on transcription regulation are even more evident in the "tet AND IPTG" gate (see Figure 5A). In this case, only the AND tetOp-lacOp configuration reproduces the AND truth table properly. Here, although the 1 output level is clearly lower than the fluorescence of the open gate (*φ* = 0.73), *σ* remains fairly high (2035 AU). In contrast, the AND lacOp-tetOp implementation presents an excess of fluorescence in correspondence to the truth table entry 01 (no tetracycline and 40 *mM* IPTG). This value is practically indistinguishable from the 1 output level measured for the 11 entry. When this gate is induced with IPTG only (01), the whole promoter regulation is due to the sole TetR whose action, however, is clearly too weak when it binds far from the TATA box. As in the YES lacOp2 case, we managed to engineer a working AND gate with the lacOp-tetOp bipartite promoter configuration via a multiple (triple) integration of the reporter-protein-encoding transcription unit (AND lacOp-tetOp-TI). In this way, the 1 output fluorescence increased highly which permitted to achieve a signal separation (2017.5 AU) comparable to the one measured for the AND tetOp-lacOp configuration (see Figure 5B). However, this implementation gives the lowest value for both *ρ* and *φ*. Moreover, a general decrease in the 0 outputs is probably imputable to an accidental multiple integration of the LacI gene (see the computational analysis below).

A remarkable fluorescence shut-off characterizes the "IPTG AND NOT(estr)" gates. Both LacI and LexA are stronger than TetR and appear only slightly more efficient when they bind close to the TATA box (they are able to repress transcription almost completely also when their operator is in proximity of the TSS–see Figure 4B). As a drawback, the 1 output level produced by these gates is rather low with *φ* equal to 0.46 in the AND lexOp-lacOp configuration and 0.31 only in the AND lacOp-lexOp one. This determines a smaller signal separation in comparison with the other AND gates in our library. In contrast, *ρ* values are moderately high, especially in the AND lacOp-lexOp where it overcomes 6.5.

A detailed summary of the performance of all the synthetic Boolean gates in our library is given in Table 1.

**Table 1:** Basic Boolean gates’ performance

**Gate**	**Input**	**1**	**max(0)**	***σ***	***ρ***	***φ***	**0/1 th.**
YES tetOp	tet	6500	1899	4601	3.42	0.96	4000
YES tetOp2	tet	4305	655	3640	6.57	1.10	2500
YES lacOp	IPTG	6190	1260	4930	4.91	1.22	3500
YES lacOp2-DI	IPTG	916	34	882	26.94	0.19	500
NOT lexOp	estr	6158	995	5163	6.19	0.93	3000
NOT lexOp2	estr	2801	164	2637	17.04	0.95	1500
AND lexOp-tetOp	tet, estr	6052.5	1641.5	4411	3.96	1.16	3000
AND tetOp-lexOp	tet, estr	4968	1245	3723	3.99	0.84	3000
AND tetOp-lacOp	tet, IPTG	2968	952	2016	3.12	0.73	2000
AND lacOp-tetOp-TI	tet, IPTG	3441.5	1424	2017.5	2.42	0.27	2250
AND lexOp-lacOp	IPTG, estr	1308	285	1023	4.90	0.46	750
AND lacOp-lexOp	IPTG, estr	1489	223	1266	6.68	0.31	900

All the fluorescence levels are expressed in arbitrary units. The last column reports the values chosen for the 0/1 threshold.

### Multiple integration: analysis

As we have seen in the previous sections, two gates did not work in their original design: the YES lacOp2 and AND lacOp-tetOp. The former showed a too strong repression from LacI (almost insensitive to high IPTG concentrations), the latter a too weak down-regulation by TetR. We managed to rescue these two gates with a multiple integration of the transcription unit expressing Citrine. This design was chosen as an alternative to a bipartite promoter with a higher transcription rate, as suggested in [12]. In the following, we show with computational argumentations that these two strategies are indeed equivalent.

For our deterministic simulations, every gate was realized with *eukaryotic* composable Parts and Pools [22] (see "Modeling" in Additional file 1). In order to compare experimental data (fluorescence) and simulation results (protein concentrations) properly, we converted both into relative quantities i.e. multiple of the output of the open YES lacOp2 and the open AND lacOp-tetOp gate.

As for the YES lacOp2 gate, we measured that the same concentration of IPTG (40 *mM*) fully inhibits LacI action on a single operator but is not effective in the two-operator case. This result might be explained by assuming a cooperative interaction among LacI proteins binding adjacent operators. Rescaling our experimental data to the output of the open YES lacOp2 gate (promoter: pLacOp2), we see that the single-lacOp-containing promoter (pLacOp) is 2.71 fold stronger and the constitutive pAct1 promoter (responsible for LacI production) even 11.25 fold more active than pLacOp2. To reproduce these proportions *in silico*, we started from the set of parameter values in [22] and changed, first, quantities such as decay rates and compartment volumes in order to reproduce the yeast cell environment more faithfully. Then we fixed an arbitrary value for the transcription initiation rate of pLacOp2 (*k*_2*ref*_ = 0.1*s*^-1^). Finally, we tuned the parameter values of reactions that play a role in transcription only since we did not consider any translation regulation mechanism in our gates’ implementation (an exhaustive list of the parameter values used in our simulations is provided in the Additional file 1).

We reproduced pLacOp experimental behavior by modifying a handful of kinetics parameter values such as the repressor-operator binding rate constant (*α*), the IPTG-LacI binding rate constant both at the promoter (*γ*) and in the nuclear LacI Pool (*λ*), the pLacOp transcription initiation rate (*k*_2_). *α* turned out to be moderately low (7.8 10^6^*M*^-1^*s*^-1^ i.e. not even ten folds higher than the RNA polymerase-DNA binding rate constant, *k*_1_ = 10^6^*M*^-1^*s*^-1^) and *λ* more than three folds lower than *γ* (i.e. IPTG was more effective on repressors bound to the promoter). This underlines that the bond between lacI and the DNA is not particularly strong. *k*_2_ was set about three folds bigger than *k*_2*ref*_ to have *in silico* the same ratio between pLacOp and pLacOp2 strength (i.e. the open gates’ relative fluorescence) as the one measured *in vivo*. This result is coherent with what we observed on each of the three single-input gates experimentally, namely a reduction of transcription efficiency in conjunction with the insertion of a second operator (i.e. by increasing the distance between the TATA box and the TSS).

**Figure 6:**
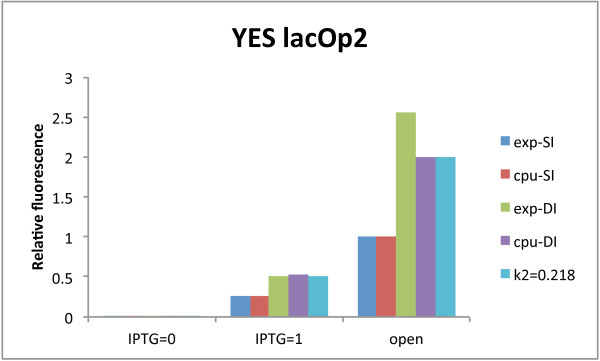
**YES lacOp2-multiple integration analysis.** Experimental data (*exp*) and computational results (*cpu*) show a good agreement concerning Citrine single and double integration both in the close (IPTG = 0 and IPTG = 1) and the open YES gate. Citrine double integration is equivalent to an increase of the transcription initiation rate (*k*_2_) of pLacOp2 from 0.1 to 0.218*s*^-1^.

We had to include in our model LacI cooperative interactions in order to mimic YES lacOp2 gate fluorescence levels. In our computational framework we had to assign to the two lac operators different affinities towards LacI molecules. The weaker operator (i.e. the one close to the TSS box) is characterized by the same binding rate constant as in the pLacOp case (*α*_*w*_ = *α*). The stronger operator shows, in contrast, an almost 10 fold higher affinity (*α*_*s*_ = 7.1 10^7^*M*^-1^*s*^-1^). When the operator close to the TATA box is taken by LacI, *α*_*w*_ is increased the to *α*_*s*_ value. Moreover, as a consequence of the stronger bond to the DNA, we had to drastically lower the value of *γ* (more than 130 folds) used for pLacOp. With this choice of parameter values, a double concentration of pLacOp2 in the closed gate configuration gives results fairly near to the experimental data (see Figure 6). Moreover, we calculated that a double integration has the same effect as an increase of pLacOp2 transcription rate from 0.1 to 0.218*s*^-1^. This proves that multiple gene integration is a valid (and easier to achieve) alternative to promoter re-engineering.

The analysis of the AND lacOp-tetOp gate required to tune *α*, *λ* and *γ* values as well. As a reference, we took the open AND lacOp-tetOp gate and set the transcription initiation rate of its promoter (pLacOpTetOp) to *k*_2*ref*_ = 0.1*s*^-1^. In this bipartite promoter configuration, the lac operator has an affinity (*α*_*lac*_ = 1.75 10^7^*M*^-1^*s*^-1^) that lies between the *α*_*w*_ and *α*_*s*_ values previously used. In order to mimic the strong LacI repression effects put in evidence by our experiments, *γ*_*lac*_ was set to a low value (100*s*^-1^). Since the tet operator appeared to be rather inefficient in binding TetR, we had to set *α*_*tet*_ value equal to the RNA polymerase binding rate constant. Moreover, both *λ*_*tet*_ and *γ*_*tet*_ were given much higher values than in the LacI case. In this way, we managed to approximate the four outputs of the original AND lacOp-tetOp gate implementation well (see Additional file 1: Table S4 and S5).

The experimental data of the AND lacOp-tetOp-TI are more difficult to explain. If, on one hand, it is clear that the Citrine gene was triple integrated, the fluorescence levels associated with each of the truth table entries are too low than the expected ones (only the 11 output is higher than the corresponding one in the original gate design). In order to reproduce this behavior, we supposed that either a single transcription factor or both of them were accidentally integrated multiply. According to our simulations, the most probable scenario is a triple integration of the LacI gene. However, with this hypothesis we were able to reproduce the gate fluorescence trend only since the computed separation between the 11 and 10 output was just half of the measured one (see Additional file 1: Table S5). Nevertheless, also in presence of an excess of LacI, the Citrine triple integration turned out to be equivalent to a higher pLacOpTetOp transcription initiation rate (*k*_2_ = 0.3166*s*^-1^) confirming the result we obtained for the YES lacOp2-DI gate.

### Assembling basic gates into circuits: distributed output architecture

All the two- and three-input circuits designed by our software arise from the composition of YES, NOT, and AND Boolean gates [17]. Therefore, digital circuits based on the sole transcription regulation can be built with the three repressor proteins (and the corresponding inducer/corepressor chemicals) we considered so far. Each circuit can be realized according either to the final gate or the distributed output architecture. Final gate schemes are, in general, more difficult to be implemented. They require more genes and more wiring among the gates with respect to their distributed output architecture counterpart. A precise gate wiring needs a more detailed promoter characterization with a better estimation of operator positional effects and leakage. Furthermore, the *fan-out*[37] of every gate has to be determined properly. Distributed output architecture, in contrast, demands that basic gates have similar performance–namely comparable output fluorescence levels–such that one can predict and clearly distinguish the 0 and 1 output of the circuit realized via gates’ composition. Since this architecture realizes an OR operation, the circuit output levels can be calculated by summing up the fluorescence measured on the basic-gate components separately. Therefore, the knowledge of basic gates’ signal separation is essential to design working distributed-output-architecture-based logic circuits.

In our library, the three one-input gates where the bipartite promoter hosts a single operator show very similar performance and can be assembled into circuits based on distributed output architecture. In particular, two-input OR gates require to integrate into the same yeast strain either two YES gates or one YES and one NOT gate (more precisely, this is an IMPLY gate in electronics’ terms) i.e 4 transcription units, one for each selective marker employed in this work (see Methods below).

**Figure 7:**
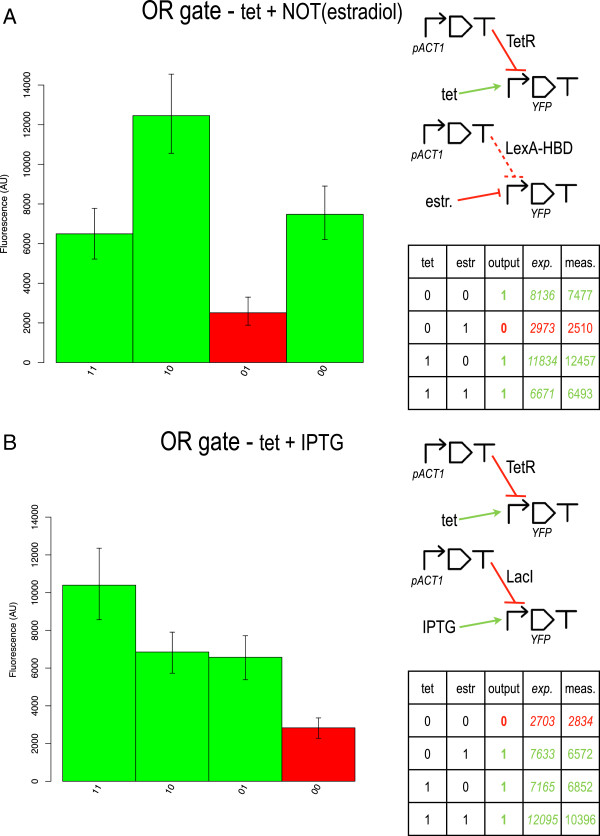
**OR gates based on distributed output architecture.** **A)** tet OR NOT(estr)–IMPLY logic function. **B)** tet OR IPTG. For both circuits, measured and expected fluorescence output levels are reported. Notice that YES tetOp gates inside both OR gates differ from the one in Table 1 since their plasmid vectors do not carry the *HIS3* marker (see Additional file 1: Table S3, for more details).

We implemented two OR gates: "tet OR NOT(estr)" and "tet OR IPTG". Both circuits show a good agreement between measured and computed fluorescence outputs (see Figure 7). Moreover, their 0/1 thresholds (4000 and 4250 AU, respectively) could be determined prior to the experiments, on the sole basis of their YES/NOT components’ digital behavior. Although, as expected, the 1 output levels are uneven and the 0 one is rather high, the significant signal separation registered from both circuits (3983 and 3738 AU, respectively) guarantees a clear reproduction of the OR gates’ truth table.

## Conclusions

In our computational work [17] we showed that complex gene digital circuits can be constructed by combining three kinds of basic Boolean gates, namely YES, NOT, and AND. Following this result, we engineered a library of 12 working gates (four YES, two NOT, and six AND) via plasmidic DNA integration into the yeast genome. Logic behavior is due the action of bacterial repressor proteins (TetR, LacI, and LexA-HDB) on synthetic bipartite promoters where repressor operators are placed between the TATA box and the TSS. By binding the DNA, repressors prevent RNA polymerases from starting transcription, a competition mechanism typical of bacterial cells. The repressors we chose are controlled by chemicals (tetracycline, IPTG, and *β*-estradiol, respectively) that represent the inputs for our digital circuits–the output is fluorescence. We characterized every gate with three main parameters: the signal separation (*σ*) between the (maximal) 0 and the (minimal) 1 output, the 1 to 0 gain (*ρ*), and the 1-factor (*φ*). *σ* and *ρ* quantify the distance between high and low fluorescence outputs and serve to place an unambiguous 0/1 threshold; *φ* specifies the efficiency of chemicals on their target protein (inducers) or points out the capability of an inactive repressor to bind the DNA in absence of its activating signal (corepressors).

With computer simulations we showed that, in order to improve the gate signal separation, multiple integration of the final gate (i.e. the transcription unit encoding for the circuit output) might be a valid alternative to the reengineering of the final gate’s promoter [12]. With this strategy we were able to rescue the only two gates that did not work in their original design. Finally, we applied the distributed output architecture [10] to genetic networks. This design strategy shows clear advantages with respect to the final gate architecture: less wiring among the gates, less genes in the network, and output levels easy to be predicted. As a proof of concept, we realized two working OR gates by the composition of either two YES gates or one YES and one NOT gate.

Every gate in our library is a small bio-sensing device and more elaborate biosensors can be built by assembling together some of these gates. New basic gates are, however, necessary in order to realize more complex digital circuits. In particular, a careful analysis of operator positional effects is required to limit promoter leakage and approach complete switch-off states. This feature is of primary importance to build circuits based on the final gate architecture that in this work was not taken into account explicitly. This family of circuits needs the wiring of three layers of Boolean gates. Numerous transcription factors–acting on several, different promoters–will be necessary and unspecific binding (gates’ cross-talk) should be avoided. TAL effectors [38] represent a possible solution to this issue since, in yeast cells, they provide a large family of orthogonal repressors/activators. Beside that, we want to extend our collection of basic Boolean gates showing a comparable digital behavior (i.e. similar *σ* values) in order to compose them into bigger distributed-output-architecture-based circuits (e.g. OR gates taking 3 or more inputs). This would permit to quantify how the performance of this design scales with the circuit size and understand possible application limits. Finally, in order to implement a higher number of circuits designed by our software [17], translational controls such as RNAi and riboswitches have to be included into our basic gates. Only at this point, we would have a complete tool for synthetic biosensing systems construction from their rational *in silico* design and simulations to their wet-lab *in vivo* implementation.

## Methods

### Plasmid vectors

Plasmids are derived from *E. coli* strain DH5 *α* [F ^-^*ϕ* 80*lacZM*15Δ (*lacZYA* - *argF*) *U*169 *deoR* *recA*1 *endA*1 *hsdR*17( $r_{K^{-}}\;m_{K^{+}}$) *phoA* *supE*44 *thi*-1 *gyrA*96 *relA*1 *λ*^-^] (Life Technologies, CA, USA). Every construct is a transcription unit (promoter–protein coding region–terminator) cloned into shuttle vectors developed in our lab (pKERG series–Gnuegge R, Rudolf F, Stelling J: unpublished). They are derivative of the pRS yeast shuttle vectors [39]. Transcription unit components are (original plasmids in brackets) : 1) yeast constitutive *ACT1* promoter (FRP337), used to lead the expression of repressor proteins; 2) synthetic bipartite promoters containing both part of the yeast *VPH1* (FRP281) and minimal *CYC1* promoter (FRP308). They always lead the production of yellow fluorescence, i.e. the circuit output; 3) TetR gene (FRP446); 4) LacI gene (FRP222); 5) LexA-HBD gene (FRP467); 6) Citrine gene (FRP677). It encodes for a reporter protein (yellow fluorescent protein-YFP); 7) *CYC1* terminator (FRP332). It represents the end of every transcription unit. Notice that both TetR and LacI are fused to a Nuclear Localization Sequence (NLS). LacI is fused to the HA tag as well. All the transcription units used in this work (reported in Additional file 1) have been implemented via the Gibson protocol [40].

### Yeast strains and plasmid integration

All yeast strains are derivative of *S. cerevisiae BY4741*[41] (*MAT***a** *his3*Δ1 *leu2*Δ*0 met15*Δ0 *ura3*Δ0 –Euroscarf, Johann Wolfgang Goethe University, Frankfurt, Germany). The starting strain for Boolean gates’ construction is FRY11 where the red fluorescent protein mKate2 [42] is constitutively expressed under *ACT1* promoter. Each Boolean gate has been implemented by integrating the corresponding transcription units into the yeast genome. All the yeast strains used in this work are listed in Additional file 1.

### Cell cultures, flow cytometry experiments, and data analysis

In order to perform flow cytometry experiments, yeast cells precultures were prepared in 5 ml YPD solution (2% peptone, 1% yeast extract, 2% glucose). They grew overnight into glass tubes at 30°C and 280 rpm. In the morning, cells cultures were approximatively 1:100 diluted into 500 *μ*l YPD. Cell solutions were poured into 2.5 ml 96-well plates. They grew at 25° C and 300 rpm for a time variable from 4 up to 8 hours. Cell cultures were then diluted again (approximatively 1:150) in YPD and induced with chemicals into an overall volume variable from 320 up to 500 *μ*l. Cells grew overnight and were re-induced with chemicals in the morning. After this last dilution (1:150, approximatively), cells were let grow for at least 4 more hours. In this way, during every flow cytometry experiment cell solution *OD*_600_ was maintained between 0.2 and 2.5 i.e. cells were in the exponential phase. This procedure was followed also to measure gates’ fluorescence in absence of chemical induction (i.e. when the only transcription unit expressing Citrine was integrated into FRY11).

Flow cytometry experiments were performed with a Becton Dickinson LSRII Fortessa analyzer equipped with a 488 nm laser (530/30 emission filter) for yellow fluorescence detection. We used 8 peaks alignment beads to guarantee a constant set-up of the machine in order to compare results from different experiments properly. In particular, we took as a reference the mean fluorescence values of the 4^*th*^ and the 6^*th*^ peak. At the beginning of each experiment, we changed the voltage of the 488 nm laser in order to place both peaks as close as possible to fixed, arbitrary positions. The 4^*th*^ peak was always located between 10080 and 10391 AU (3.1*%* variability with respect to the minimum value) and the 6^*th*^ peak between 72208 and 74165 AU (2.7*%*). Moreover, we registered these fluorescence levels also at the end of every analysis to assure that the machine set-up did not change considerably over a single experiment (see Additional file 1: Figure S3).

We recorded 10000 events for every sample. All the data were analyzed with R by using the flowcore (Bioconductor) package. Since fluorescence data follow a log-normal distribution, we took the distribution *median* to quantify the gate output. Coherently, we placed the experimental error bars at the first and third distribution quartile. Finally, both fluorescence median and errors were subtracted the background fluorescence i.e. the the yellow fluorescence signal detected on FRY11 (where the Citrine gene was not integrated).

### Chemicals

We used three different chemicals in our experiments: tetracycline, IPTG, and *β*-estradiol (Sigma-Aldrich). We prepared 10 *mM* tetracyline stock solution in ethanol, 1*M* IPTG stock solutions in water, and 10 *mMβ*-estradiol stocks in ethanol. From their stocks, chemicals were diluted in YPD for flow cytometry experiments.

## Competing interests

I declare I have no competing interests.

## Supplementary Material

Additional file 1 Supplementary Material. Click here for file
