# Supplementary material for: In silico design and in vivo implementation of yeast gene Boolean gates
Source: J Biol Eng. 2014 Feb 2;8:6. doi: 10.1186/1754-1611-8-6 (PMC3926364; doi:10.1186/1754-1611-8-6)
Supplement: Additional file 1 — Supplementary Material. [file 1754-1611-8-6-S1.pdf]

In silico design and *in vivo* implementation of yeast gene Boolean gates

Additional file 1

Supplementary Material

Mario A. Marchisio[1,2]

[1] Department of Biosystems Science and Engineering (D-BSSE), ETH Zurich, Mattenstrasse 26, 4058 Basel, Switzerland

[2] Current address: School of Life Science and Technology, Harbin Institute of Technology (HIT), 2 Yikuang Street, Nan Gang District, 150080 Harbin, P. R. China

email: marchisio@hit.edu.cn

Introduction

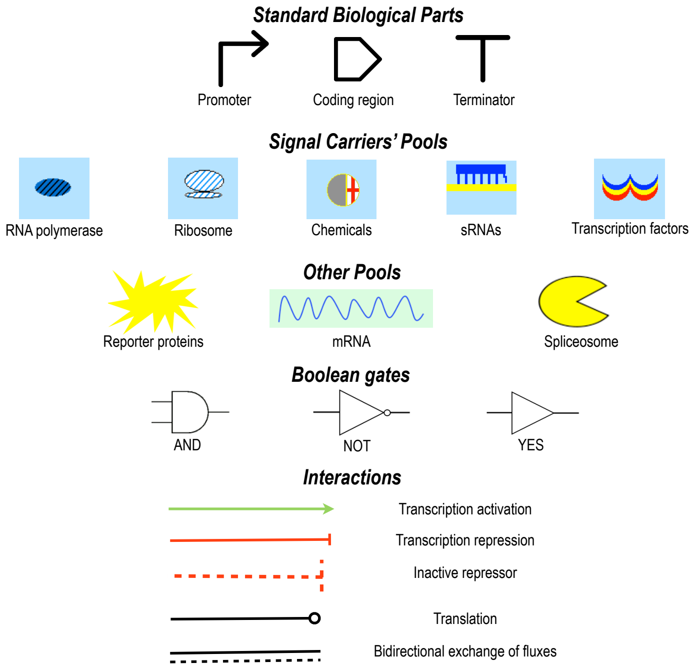

Figure S1: **Symbols.** Summary of the symbols used in this work.

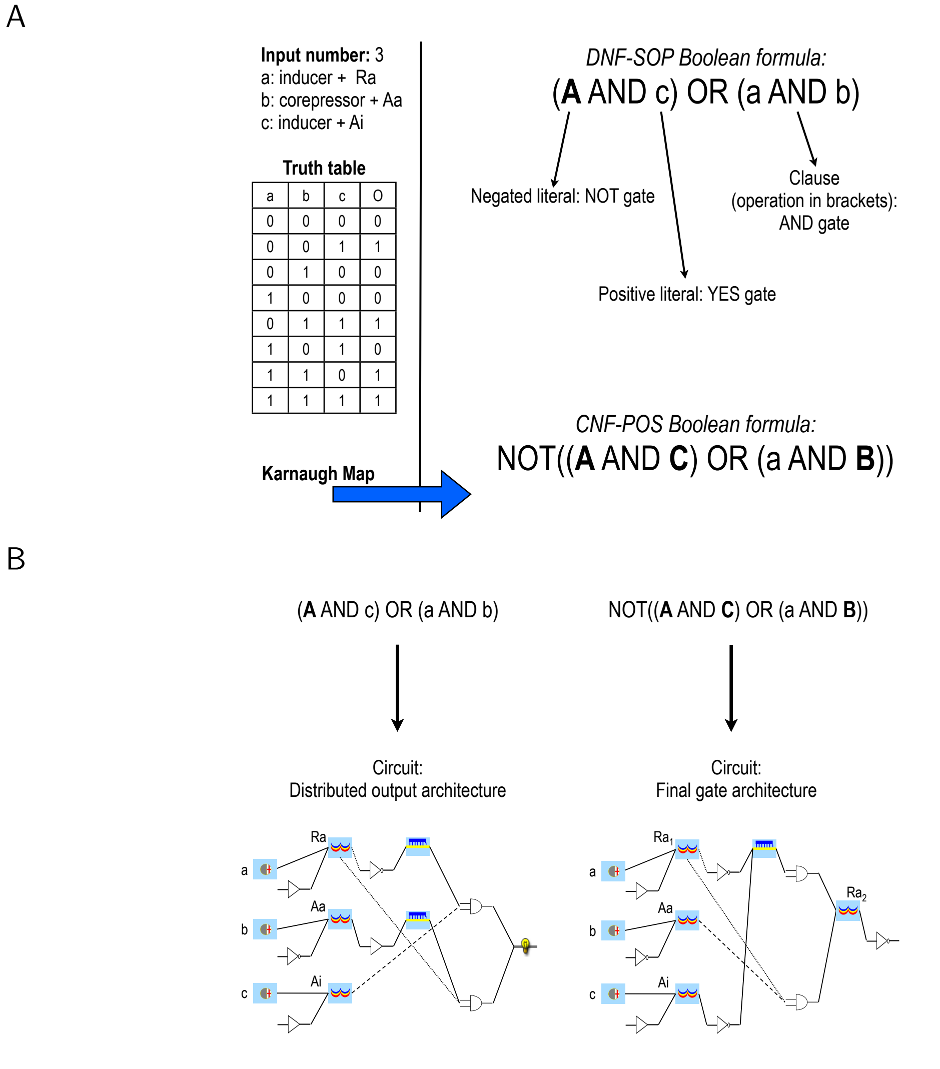

Figure S2: **Automatic gene digital circuit design.** A) Our software requires: a truth table, the input chemicals (inducers/corepressors), and chemicals' targets (in the figure: Ra, active repressor; Aa, active activator; Ai, inactive activator). The truth table is converted both into the DNF-SOP and the CNF-POS formulas via the Karnaugh map method. B) Formulas are translated into circuits organized into two (distributed output architecture) or three (final gate architecture) layers of gates and Pools of common signal carriers.

Materials and Methods

Plasmid name	Construct	Selective Marker
URA3 FRP908	pAct1-tetR-NLS-Cyc1term	*URA3*
FRP981	pAct1-lacI-NLS-HAtagGeneva-Cyc1term	*LEU2*
FRP810	pAct1-lexADBD-HBD-Cyc1term	*MET15*
FRP1022	pAct1-lexADBD-HBD-Cyc1term	*URA3*
FRP1115	pAct1-lacI-NLS-HAtagGeneva-Cyc1term	*MET15*
FRP920	pVph1-tetOp-pCyc1min-YFP-Cyc1term	*HIS3*
FRP827	pVph1-tetOp2-pCyc1min-YFP-Cyc1term	*HIS3*
FRP966	pVph1-lacOp-pCyc1min-YFP-Cyc1term	*HIS3*
FRP970	pVph1-lacOp2-pCyc1min-YFP-Cyc1term	*HIS3*
FRP1016	pVph1-lexOp-pCyc1min-YFP-Cyc1term	*HIS3*
FRP1021	pVph1-lexOp2-pCyc1min-YFP-Cyc1term	*HIS3*
FRP1017	pVph1-lexOp-tetOp-pCyc1min-YFP-Cyc1term	*HIS3*
FRP1018	pVph1-tetOp-lexOp-pCyc1min-YFP-Cyc1term	*HIS3*
FRP1019	pVph1-lexOp-lacOp-pCyc1min-YFP-Cyc1term	*HIS3*
FRP1020	pVph1-lacOp-lexOp-pCyc1min-YFP-Cyc1term	*HIS3*
FRP963	pVph1-tetOp-lacOp-pCyc1min-YFP-Cyc1term	*HIS3*
FRP964	pVph1-lacOp-tetOp-pCyc1min-YFP-Cyc1term	*HIS3*
FRP1142	pVph1-tetOp-pCyc1min-YFP-Cyc1term	*LEU2*
FRP1190	pVph1-tetOp-pCyc1min-YFP-Cyc1term	*MET15*

Table S1: **List of the plasmids** employed in the assembly of Boolean gates. In every plasmid a transcription unit is delimited by the restriction sites XbaI and KpnI.

VPH1 promoter sequence

TCTAGAAAGTGAAGAGACAAATTTATATAGTTATAGAATAAATATCAGATAGATAAGAAA
AAAAAATTAGTTAAACATTAATATATATATATGTGTAGTGACTGACATACGTATGACTGCT
AGTAATCCAGTTGCCGAGCTATTGTTGCAGATTGAAATGTCATCACGTGGACATTATAT
CATCATTAACGACTAATGCGTCAAAAAAAAATGGGCTAAAAAAAAAAAAAGCAAAAAA
AAGAAACTCAGATAAGTCGATATGGAAAACCTTTTTTCTTGAGTGCAACCCTTTTAGAG
GTTACAAAACAACCGGT

minimal *CYC1* promoter sequence

TTCTTTCCTTATACATTAGGACCTTTGCAGC**A**TAAATTACTATACTTCTAT

Strain name	Genotype	Gate
FRY630	FRY11 FRP920::HIS3	*open* YES tetOp
FRY651	FRY11 FRP920::HIS3 FRP908::URA3	YES tetOp
FRY511	FRY11 FRP827	*open* YES tetOp2
FRY605	FRY11 FRP827::HIS3 FRP908::URA3	YES tetOp2
FRY697	FRY11 FRP966::HIS3	*open* YES lacOp
FRY872	FRY11 FRP966::HIS3 FRP981::LEU2	YES lacOp
FRY713	FRY11 FRP970::HIS3	*open* YES lacOp2
FRY854	FRY11 FRP970::HIS3 FRP981::LEU2	YES (lacOp2)
FRY714	FRY11 FRP970^2::HIS3	*open* YES lacOp2-DI
FRY1052	FRY11 FRP970^2::HIS3 FRP981::LEU2	YES lacOp2-DI
FRY795	FRY11 FRP1016::HIS3	*open* NOT lexOp
FRY819	FRY11 FRP1016::HIS3 FRP810::MET15	NOT lexOp
FRY797	FRY11 FRP1021::HIS3	*open* NOT lexOp2
FRY821	FRY11 FRP1021::HIS3 FRP810::MET15	NOT lexOp2
FRY799	FRY11 FRP1017::HIS3	*open* AND lexOp-tetOp
FRY831	FRY11 FRP1017::HIS3 FRP908::URA3 FRP810::MET15	AND lexOp-tetOp
FRY801	FRY11 FRP1018::HIS3	*open* AND tetOp-lexOp
FRY832	FRY11 FRP1018::HIS3 FRP908::URA3 FRP810::MET15	AND tetOp-lexOp
FRY677	FRY11 FRP963::HIS3	*open* AND tetOp-lacOp
FRY851	FRY11 FRP963::HIS3 FRP908::URA3 FRP981::LEU2	AND tetOp-lacOp
FRY679	FRY11 FRP964::HIS3	*open* AND lacOp-tetOp
FRY853	FRY11 FRP964::HIS3 FRP908::URA3 FRP981::LEU2	AND lacOp-tetOp
FRY680	FRY11 FRP964^3::HIS3	*open* AND lacOp-tetOp-TI
FRY968	FRY11 FRP964^3::HIS3 FRP908::URA3 FRP981::LEU2	AND lacOp-tetOp-TI
FRY802	FRY11 FRP1019::HIS3	*open* AND lexOp-lacOp
FRY856	FRY11 FRP1019::HIS3 FRP1022::URA3 FRP981::LEU2	AND lexOp-lacOp
FRY803	FRY11 FRP1020::HIS3	*open* AND lacOp-lexOp
FRY977	FRY11 FRP1020::HIS3 FRP1022::URA3 FRP1115::MET15	AND lacOp-lexOp
FRY1041	FRY11 FRP1142::LEU2 FRP908::URA3	YES tetOp
FRY1089	FRY11 FRP1142::LEU2 FRP908::URA3 FRP1016::HIS3 FRP810::MET15	OR (tetOp+lexOp)
FRY1110	FRY11 FRP1190::MET15 FRP908::URA3	YES tetOp
FRY1109	FRY11 FRP1190::MET15 FRP908::URA3 FRP966::HIS3 FRP981::LEU2	OR (tetOp+lacOp)

Table S2: **List of yeast strains** whose genome was modified via gene Boolean gate's integration. Upper indexes indicate multiple integrations. Plasmids are described in Table 1.

A

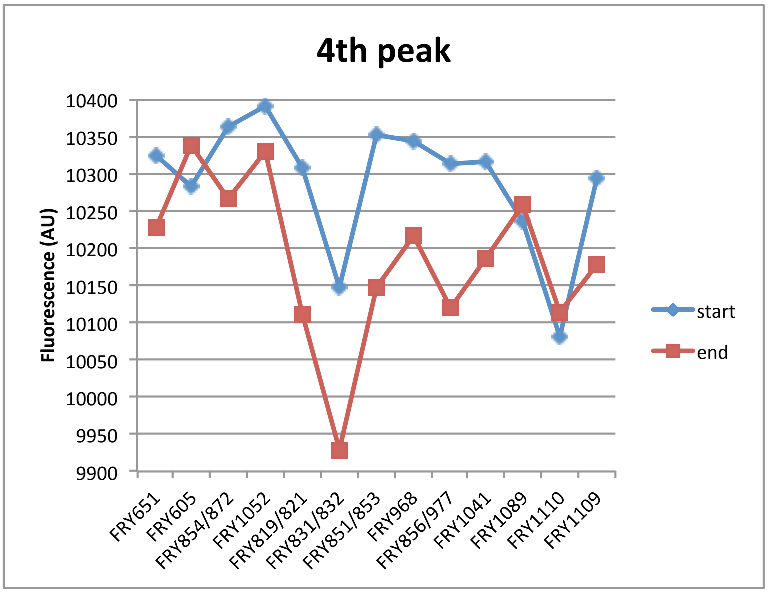

B

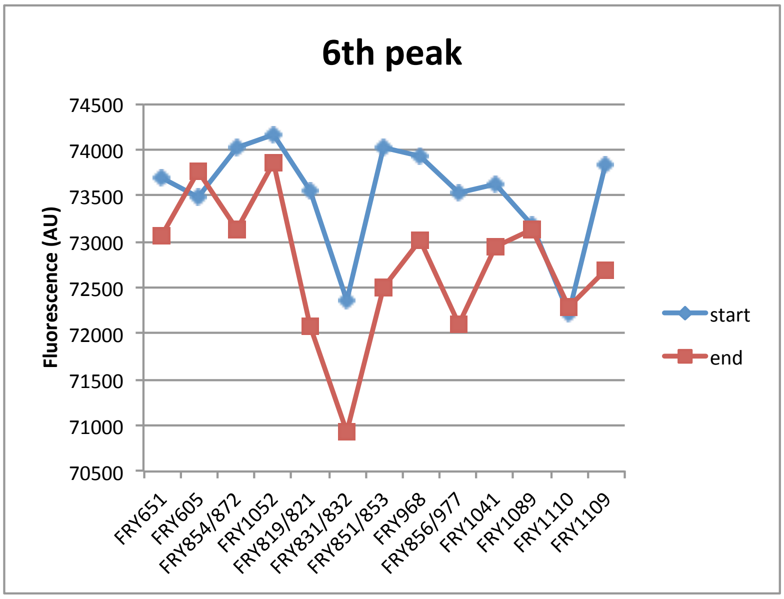

Figure S3: 8 **peaks alignment beads fluorescence.** For every experiment (labelled with one or two yeast strain corresponding to closed gates) the initial and final values of the mean fluorescence of the 4th (A) and the 6th (B) peak are reported. Within a single experiment, the biggest variation we observed was of 2.1% (4th peak) and 2.0% (6th peak) of the initial mean fluorescence value.

Experimental results

Three different YES tetOp gate implementation

Strain name	Marker	1	max(0)	σ	ρ	φ	0/1 th.
FRY651	*HIS3*	6500	1899	4601	3.42	0.96	4000
FRY1041	*LEU2*	5675.5	1978	3697.5	2.87	0.90	4000
FRY1110	*MET15*	5904.5	1443	4461.5	4.09	1.20	4000

Table S3: **Comparison of the performance** of the three YES tetOp gates implemented in this work. Fluorescence levels are expressed in arbitrary units. The last column refers to the chosen low/high output threshold.

A

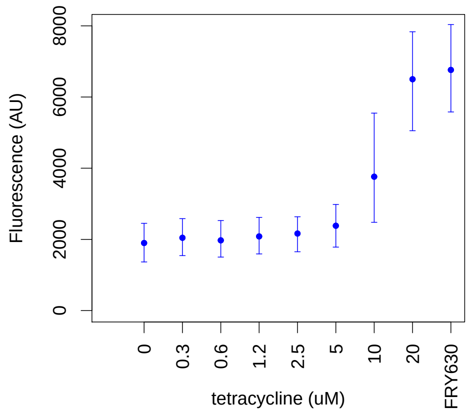

B

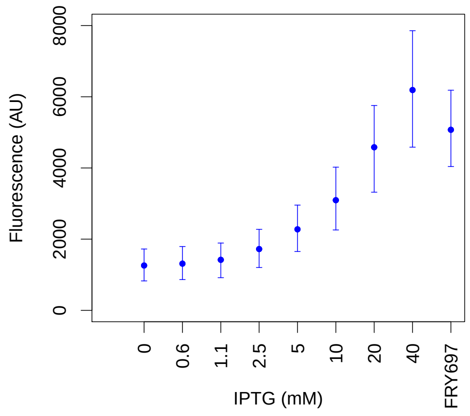

C

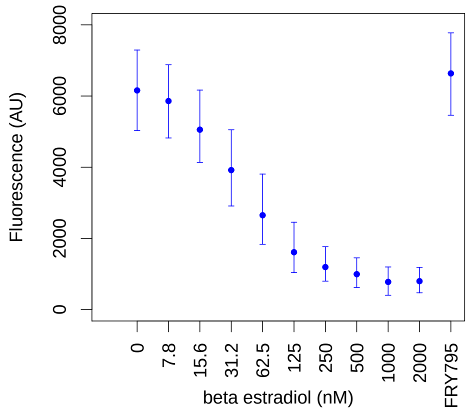

Figure S4: **Titration curves.** A) tetracycline-TetR (YES tetOp gate). B) IPTG-LacI (YES lacOp gate). c) β-estradiol–LexA-HBD (NOT lexOp gate). For each curve, the corresponding open gate–indicated with the yeast strain name–was taken as positive control.

Computational analysis

YES lacOp2-DI analysis

YES gate	IPTG=0	IPTG=1	open
lacOp	0.67	3.31	2.71
lacOp*	0.67	2.68	2.71
lacOp2	0.01	0.26	1.00
lacOp2* (k_{2ref}=0.1)	0.01	0.26	1.00
lacOp2-DI	0.02	0.50	2.56
lacOp2-DI*	0.02	0.53	2.00
lacOp2* (k_2=0.218)	0.02	0.50	2.00

Table S4: **Comparison of experimental and computational (*) relative fluorescence** of YES lacOp, YES lacOp2, and YES lacOp2-DI gates.

AND lacOp-tetOp-TI analysis

AND gate	00	01	10	11	open
lacOp-tetOp	0.10	0.42	0.14	0.51	1.00
lacOp-tetOp* (k_{2ref}=0.1)	0.07	0.43	0.10	0.55	1.00
lacOp-tetOp-TI	0.00	0.35	0.12	0.85	3.12
lacOp-tetOp TI*	0.20	1.28	0.30	1.64	3.00
lacOp-tetOp-TI with lacI-TI*	0.07	0.69	0.10	0.94	3.00
lacOp-tetOp with lacI-TI* (k_2=0.3166)	0.06	0.62	0.09	0.86	3.00

Table S5: **Comparison of experimental and computational (*) relative fluorescence** of AND lacOp-tetOp, and AND lacOp-tetOp-TI. The first input digit refers to tetracycline, the second one to IPTG.

Parameter values

VOLUME	Value	Reference
v_{cell}	$42 \cdot 10^{-15} l$	[4]
$v_{nucleus}$	$2.9 \cdot 10^{-15} l$	7% v_{cell} [3]
$v_{cytoplasm}$	$39.1 \cdot 10^{-15} l$	

Protein decay rates k_{dp} is the same for free and DNA-bound proteins. Their values are listed below (nuclear protein Pools).
Notice that k_{el} = gene length/v_{pol} and k_{el}^r = gene length/v_{rib}. The mRNA decay rate k_d is defined by the terminator.

POOLS	Value	Reference
pol^{free}	5000	arbitrary
Y^{free}	5000	arbitrary
rib^{free}	23000	arbitrary

PROMOTERS-common	Value	Reference
k_1	$10^6 M^{-1}s^{-1}$	RNA polymerase-promoter binding rate constant [6]
k_{-1}	$1s^{-1}$	RNA polymerase-promoter unbinding rate [6]
k_2^{lk}	$5\,10^{-5}s^{-1}$	transcription initiation rate due to leakage[6]

PROMOTER-pAct1	Value	Reference
k_2	$1.53s^{-1}$	transcription initiation rate

PROMOTER-pLacOp	Value	Reference
k_2	$0.284s^{-1}$	transcription initiation rate
α	$7.8\,10^6 M^{-1}s^{-1}$	LacI-DNA binding rate constant
β	$9s^{-1}$	LacI-DNA unbinding rate
γ	$6800M^{-1}s^{-1}$	IPTG-LacI (on the DNA) binding rate constant

PROMOTER-pLacOp2	Value	Reference
k_2	$0.1s^{-1}$	transcription initiation rate
α_s	$7.1\,10^7 M^{-1}s^{-1}$	LacI-strong operator binding rate constant
α_w	$7.8\,10^6 M^{-1}s^{-1}$	LacI-weak operator binding rate constant
α_c	$7.1\,10^7 M^{-1}s^{-1}$	LacI-weak operator binding rate constant with cooperativity
β_s	$9s^{-1}$	LacI-strong operator unbinding rate
β_w	$224s^{-1}$	LacI-weak operator unbinding rate
β_c	$20s^{-1}$	LacI-weak operator unbinding rate with cooperativity
γ	$50M^{-1}s^{-1}$	IPTG-LacI (on the DNA) binding rate constant

PROMOTER-pLacOpTetOp	Value	Reference
k_2	$0.1s^{-1}$	transcription initiation rate
α_{lac}	$1.75\,10^7 M^{-1}s^{-1}$	LacI-DNA binding rate constant
α_{tet}	$10^6 M^{-1}s^{-1}$	TetR-DNA binding rate constant
β_{lac}	$9s^{-1}$	LacI-DNA unbinding rate
β_{tet}	$9s^{-1}$	TetR-DNA unbinding rate
γ_{lac}	$100M^{-1}s^{-1}$	IPTG-LacI (on the DNA) binding rate constant
γ_{tet}	$10^4 M^{-1}s^{-1}$	tetracycline-TetR (on the DNA) binding rate constant

CODING REGIONS and mRNA Pools	Value	Reference
YFP length	$726\,nt$	
LacI length	$1208\,nt$	
TetR length	$684\,nt$	
v_{pol}	$23.3\,nt/s$	polymerase speed [5]
v_{rib}	$24\,nt/s$	ribosome speed [5]
k_{1y}	$1500M^{-1}s^{-1}$	spliceosome-mRNAbinding rate constant [5]
k_{-1y}	$0.0017s^{-1}$	spliceosome-mRNA unbinding rate [5]
k_{2y}	$0.033s^{-1}$	splicing rate [5]
k_m	$0.00055s^{-1}$ (30min)	mRNA maturation rate [5]
k_{1r}	$10^6 M^{-1}s^{-1}$	ribosome-mRNA binding rate constant [5]
k_{-1r}	$0.01s^{-1}$	ribosome-mRNA unbinding rate [5]
k_{2r}	$0.02s^{-1}$	translation initiation rate [5]
ζ_r	$0.5s^{-1}$	protein synthesis rate [5]
k_{tr}	$8.3 \cdot 10^{-3}s^{-1}$ (2 min)	nuclear import rate [5]

TERMINATORS	Value	Reference
k_d	$5.7\,10^{-4}s^{-1}$ 20.1 min	mRNA decay rate [9]
ζ	$31.25s^{-1}$	RNA polymerase-DNA unbinding rate [5]

Repressor POOLS-common	Value	Reference
k_{dp}	$2.7\,10^{-4}s^{-1}$ (43 min)	decay rate [1]
δ	$10^9M^{-1}s^{-1}$	dimerization rate constant [6]
ϵ	$10s^{-1}$	dimer separation rate [6]

LacI POOL	Value	Reference
λ	$2180M^{-1}s^{-1}$	IPTG-LacI binding rate constantl [6]
μ	$10s^{-1}$	IPTG-LacI unbinding rate [6]

TetR POOL	Value	Reference
λ	$10^6M^{-1}s^{-1}$	tetracycline-TetR binding rate constantl [6]
μ	$10s^{-1}$	tetracycline-TetR unbinding rate [6]

tetracycline POOL	Value	Reference
s^{free}	$0/20\,10^{-6}M$	initial concentrations

IPTG POOL	Value	Reference
s^{free}	$0/0.04M$	initial concentrations

Modeling

In silico, both YES lacOp2 and AND lacOp-tetOp gate have been realized by composing eukaryotic Parts and Pools [7]. Composable Parts are DNA segments with a well-defined function either in transcription or translation [2]; Pools are the hypothetical places where *free* molecules (i.e. not bound to the DNA or the mRNA) are stored. Eukaryotic Parts and Pools present substantial differences from the bacterial ones presented in [6]: 1) Promoters, Coding Regions (for proteins and small RNAs), and Terminators are the only types of Parts that make up a circuit, ribosome binding sites (RBS) are no longer present ; 2) mature mRNAs corresponding to different genes are stored into separate Pools; 3) mRNA maturation is taken into account and a spliceosome Pool is used; 4) RNA interference replaces simple base pairing between small RNAs and mRNA; 5) Parts and Pools are placed into two communicating compartments: nucleus and cytoplasm. However, the principles of modeling and designing synthetic gene circuits with eukaryotic composable Parts and Pools are the same as in bacterial cells. Parts and Pools are described independently via full mass-action kinetics. This allows to define an interface made of fluxes of Common Signal Carriers and other molecules. Common Signal Carriers are RNA polymerases (flux: PoPS, Polymerases Per Second), ribosomes (RiPS, Ribosomes Per Second), transcription factors (FaPS, Factors Per Second), small RNAs (RNAPS, RNAs Per Second), and chemicals (SiPS: Signals Per Second). PoPS and RiPS fluxes are either the input/output of Parts (the former along the DNA, the latter on the mRNA) or they are exchanged between the Polymerase Pool and each promoter in the nucleus (PoPS^b) or the Ribosome Pool and any mRNA Pool in the cytoplasm (RiPS^b) . The index b stands for *balance* and indicates a bidirectional communication. FaPS and RNAPS fluxes allow interactions among transcription units whereas SiPS is a currency exchanged by the whole circuit and the environment.

YES lacOp2

Figure shows the schematic for the YES lacOp2 gate with eukaryotic Parts and Pools. The two boxes in the nucleus are the transcription units employed in the circuit: one producing LacI and the other Citrine i.e. the yellow fluorescent protein (YFP). Graphically, the LacI Pool in the nucleus plays the role of an interface between the two transcription units and the IPTG (isopropil-β-D-1-tiogalattopiranoside) Pool outside the cell is the interface between the Boolean gate and the environment. In the following, we give a model for each gate's Part and Pool where a list of biochemical reactions is accompanied with the calculation of the fluxes received or sent by the circuit component. Notice that the symbol $\Longrightarrow$ means that a flux of molecules ends entirely into a species.

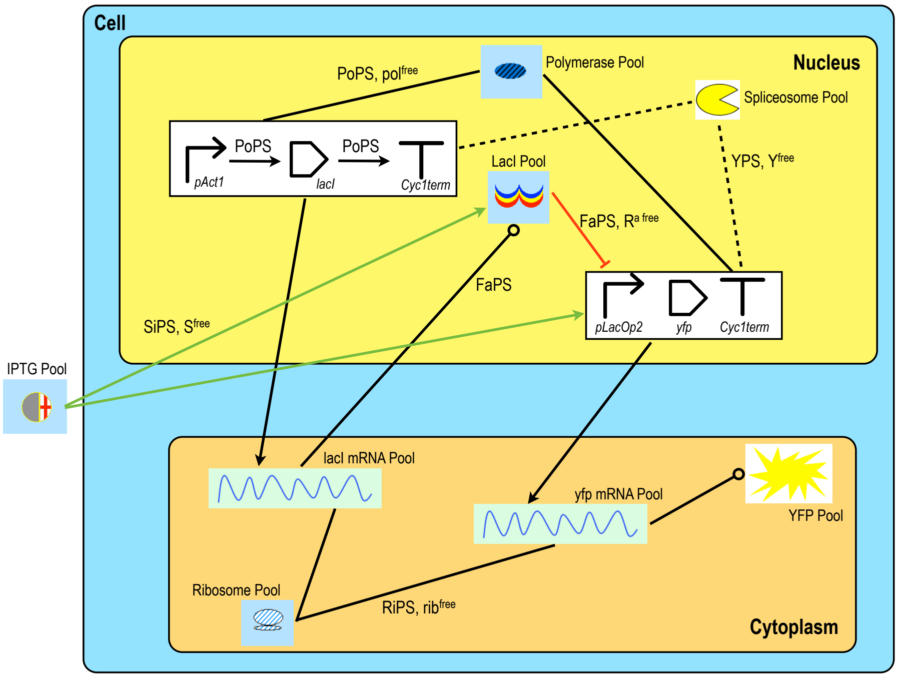

Figure S5: **YES lacOp2 scheme.** Schematic of the Boolean gate YES lacOp2. RNA Polymerase Pool is connected to both transcription units. In particular, this Pool exchanges a $PoPS^b$ flux with each promoter and receives a $PoPS^{in}$ flux from every terminator (for the sake of simplicity, we traced a single straight line between the Pool and each transcription unit and indicated as $PoPS$ the information exchanged). The two promoters are also constantly informed (during a simulation) about the amount of RNA Polymerase available in the Pool (pol^{free}). An analogous description holds for the ribosome Pool (double connection to each mRNA Pool) whereas the spliceosome Pool has a single link to every Coding region. The IPTG Pool is connected to both the LacI Pool and the pLacOp2 promoter since IPTG can bind and inactivate LacI molecules both on the DNA and far from it. Therefore, the LacI Pool–which receives a flux of proteins ($FaPS^{in}$ from the cytoplasm–exchange with the pLacOp2 promoter a flux of active ($FaPS_a^b$) and inactive ($FaPS_i^b$) LacI and informs the promoter with the amount of currently available active repressors (R^a). Finally, the YFP Pool permits to read the circuit output during a computer simulation.

Constitutive pAct1 promoter

Species and fluxes

$p0^f$	free promoter (RNA polymerase binding site)
$p0^t$	promoter *taken* by RNA polymerases
Pol^{free}	RNA polymerases available in the Polymerase Pool
Pol^{cl}	RNA polymerase in the promoter cleaning phase. Sent as $PoPS^{out}$ to the lacI coding region
$PoPS^b$	exchanged with the Polymerase Pool
$PoPS^{out}$	sent to LacI coding region

Notice that Pol^{cl} is a fictitious species [8] since it does not appear explicitly into the circuit SBML file but it is replaced by the $PoPS^{out}$ flux.

Reactions

$$\begin{aligned} Pol^{free} + p0^f \longleftrightarrow p0^t & \qquad k_1, k_{-1} \\ p0^t \longrightarrow p0^f + Pol^{cl} & \qquad k_2 \end{aligned}$$

Fluxes calculation

$$\begin{aligned} PoPS^{out} &= k_2 p0^t \\ PoPS^b &= k_1 Pol^{free} p0^f - k_{-1} p0^t \end{aligned}$$

LacI and YFP coding region

Species and fluxes

$PoPS^{in}$	from the adjacent promoter
Y^{free}	available spliceosome molecules into their Pool
$[PolA]$	RNA polymerase bound to the DNA before starting the elongation phase
Pol^{el}	RNA polymerase in the promoter elongation phase. It is a fictitious species replaced by $PoPS^{out}$
u_{mRNA}	unspliced mRNA
$[Yu_{mRNA}]$	spliceosome molecules bound to u_{mRNA}
n_{mRNA}	nuclear mRNA
m_{mRNA}	mature mRNA. It is a fictitious species replaced by $RNAPS^{out}$
$RNAPS^{out}$	flux of mature mRNA sent to the corresponding mRNA Pool in the cytoplasm
$PoPS^{out}$	sent to the adjacent terminator

Reactions

$$\begin{aligned} PoPS^{in} \Longrightarrow [PolA] & \\ [PolA] \longrightarrow u_{mRNA} + Pol^{el} & \qquad k_{el} \\ Y^{free} + u_{mRNA} \longleftrightarrow [Yu_{mRNA}] & \qquad k_{1y}, k_{-1y} \\ [Yu_{mRNA}] \longrightarrow Y^{free} + n_{mRNA} & \qquad k_{2y} \\ n_{mRNA} \longrightarrow m_{mRNA} & \qquad k_m \\ u_{mRNA} \longrightarrow & \qquad k_d \end{aligned}$$

$$
\begin{array}{ll}
n_{mRNA} \longrightarrow & k_d \\
[Yu_{mRNA}] \longrightarrow Y^{free} & k_d
\end{array}
$$

Fluxes calculation

$$
\begin{aligned}
RNAPS^{out} &= k_m n_{mRNA} \\
PoPS^{out} &= k_{el}[PolA] \\
YPS^b &= k_{1y}Y^{free}u_{mRNA} - (k_{-1y} + k_{2y} + k_d)[Yu_{mRNA}]
\end{aligned}
$$

Cyc1 terminator

Species and fluxes

$PoPS^{in}$	from the adjacent coding region
$[PolT]$	RNA polymerase bound to the terminator
Pol^{free}	RNA polymerase leaving the DNA. Sent to the Polymerase Pool as $PoPS^{out}$
$PoPS^{out}$	sent to the Polymerase Pool

Reactions

$$
\begin{array}{ll}
PoPS^{in} \Longrightarrow [PolT] & \\
[PolT] \longrightarrow Pol^{free} & \zeta
\end{array}
$$

Fluxes calculation

$$
PoPS^{out} = \zeta[PolT]
$$

The LacI and YFP mRNA Pool

Species and fluxes

$RiPS^b$	exchanged with the ribosome Pool
$RNAPS^{in}$	flux of mature mRNA from the corresponding coding region
$PoPS^{in}_{lk}$	flux of RNA polymerase due to a promoter leakage
r_0^f	free mature mRNA
rib^{free}	free ribosomes available in their Pool
r_0^t	mature mRNA *taken* by ribosomes
$[ribSTART]$	ribosomes bound to the START codon
$[ribSTOP]$	ribosomes bound to the STOP codon
$protein_c$	proteins in the cytoplasm
$protein_n$	proteins in the nucleus. It is a fictitious species
$RiPS^{out}$	sent to the ribosome Pool
$FaPS^{out}$	LacI or YFP flux sent to the corresponding Pool in the nucleus

Reactions

$$
\begin{aligned}
RNAPS^{in} &\Longrightarrow r_0^f \\
PoPS_{lk}^{in} &\Longrightarrow r_0^f && \text{(only for YFP)} \\
rib^{free} + r_0^f &\longleftrightarrow r_0^t && k_{1r}, k_{-1r} \\
r_0^t &\longrightarrow r_0^f + [ribSTART] && k_{2r} \\
[ribSTART] &\longrightarrow [ribSTOP] && k_{el}^r \\
[ribSTOP] &\longrightarrow rib^{free} + protein_c && \zeta_r \\
r_0^f &\longrightarrow && k_d \\
r_0^t &\longrightarrow rib^{free} && k_d \\
protein_c &\longrightarrow && k_{dp} \\
protein_c &\longrightarrow protein_n && k_{tr}
\end{aligned}
$$

Fluxes calculation

$$
\begin{aligned}
RiPS^b &= k_{1r} rib^{free} r_0^f - k_{-1r} r_0^t \\
RiPS^{out} &= \zeta_r [ribSTOP] + k_d r_0^t \\
FaPS^{out} &= k_{tr} protein_c
\end{aligned}
$$

The LacI Pool

Species and fluxes

$FaPS^{in}$	from the corresponding mRNA Pool in the cytoplasm
R^m	LacI monomers
R^a	LacI dimers (active i.e. not bound to IPTG)
R^i	LacI dimers (inactive i.e. bound to IPTG)
S^{free}	available IPTG molecules in their corresponding Pool
$SiPS^b$	exchanged with the IPTG Pool

Reactions

$$
\begin{aligned}
FaPS^{in} &\Longrightarrow R^m \\
2R^m &\longleftrightarrow R^a && \delta,\ \epsilon \\
R^m &\longrightarrow && k_{dp} \\
R^a &\longrightarrow && k_{dp} \\
S^{free} + R^a &\longleftrightarrow R^i && \lambda, \mu \\
R^i &\longrightarrow S^{free} && k_{dp}
\end{aligned}
$$

IPTG is supposed to bind only LacI dimers.

Fluxes calculation

$$
SiPS^b = \lambda S^{free} R^a - (\mu + k_{dp}) R^i
$$

Regulated pLacOp2 promoter

Species and fluxes

Pol^{free}	RNA polymerases available in their Pool
R^a	free active LacI molecules available in their Pool
R^i	free inactive LacI molecules
S^{free}	IPTG molecules available in their Pool
$p0^f O_1^f O_2^f$	completely free promoter
$p0^t O_1^f O_2^f$	RNA polymerase bound to the promoter ($p0$ is the polymerase binding site)
$p0^f O_1^t O_2^f$	LacI bound to the strong operator O_1 (close to the TATA box)
$p0^f O_1^f O_2^t$	LacI bound to the weak operator O_2 (close to the TSS)
$p0^f O_1^t O_2^t$	LacI bound to both operators
Pol^{cl}	RNA polymerase in the promoter cleaning phase
Pol_{lk}^{cl}	RNA polymerase in the cleaning phase due to promoter leakage. This is a fictitious species
$PoPS^b$	exchanged with the polymerase Pool
$PoPS^{out}$	sent to the YFP coding region
$PoPS_{lk}^{out}$	sent to the YFP mRNA Pool in the cytoplasm
$FaPS_a^b$	flux of active LacI exchanged with the LacI Pool
$FaPS_i^{out}$	flux of inactive LacI sent to the LacI Pool
$SiPS^{in}$	from the IPTG Pool

Reactions

$$
\begin{aligned}
Pol^{free} + p0^f O_1^f O_2^f &\longleftrightarrow p0^t O_1^f O_2^f && k_1, k_{-1} \\
p0^t O_1^f O_2^f &\longrightarrow p0^f O_1^f O_2^f + Pol^{cl} && k_2 \\
R^a + p0^f O_1^f O_2^f &\longleftrightarrow p0^f O_1^t O_2^f && \alpha_s, \beta_s \\
R^a + p0^f O_1^f O_2^t &\longleftrightarrow p0^f O_1^t O_2^t && \alpha_s, \beta_s \\
R^a + p0^f O_1^f O_2^f &\longleftrightarrow p0^f O_1^f O_2^t && \alpha_w, \beta_w \\
R^a + p0^f O_1^t O_2^f &\longleftrightarrow p0^f O_1^t O_2^t && \alpha_c, \beta_c \\
S^{free} + p0^f O_1^t O_2^f &\longrightarrow p0^f O_1^f O_2^f + R^i && \gamma \\
S^{free} + p0^f O_1^t O_2^t &\longrightarrow p0^f O_1^f O_2^t + R^i && \gamma \\
S^{free} + p0^f O_1^f O_2^t &\longrightarrow p0^f O_1^f O_2^f + R^i && \gamma \\
S^{free} + p0^f O_1^t O_2^t &\longrightarrow p0^f O_1^t O_2^f + R^i && \gamma \\
p0^f O_1^t O_2^f &\longrightarrow p0^f O_1^f O_2^f && k_{dp} \\
p0^f O_1^t O_2^t &\longrightarrow p0^f O_1^f O_2^t && k_{dp} \\
p0^f O_1^f O_2^t &\longrightarrow p0^f O_1^f O_2^f && k_{dp} \\
p0^f O_1^t O_2^t &\longrightarrow p0^f O_1^t O_2^f && k_{dp}
\end{aligned}
$$

Fluxes calculation

$$
\begin{aligned}
PoPS^{out} &= k_2 p0^t O_1^f O_2^f \\
PoPS_{lk}^{out} &= k_2^{lk} (p0^f O_1^f O_2^t + p0^f O_1^t O_2^f + p0^f O_1^t O_2^t) \\
PoPS^b &= k_1 Pol^{free} p0^f O_1^f O_2^f - k_{-1} p0^t O_1^f O_2^f
\end{aligned}
$$

$$
\begin{aligned}
FaPS_a^b &= \alpha_s R^a (p0^f O_1^f O_2^f + p0^f O_1^f O_2^t) + \\
&+ \alpha_w R^a p0^f O_1^f O_2^f + \alpha_c R^a p0^f O_1^t O_2^f + \\
&- \beta_s (p0^f O_1^t O_2^f + p0^f O_1^t O_2^t) - \beta_w p0^f O_1^f O_2^t + \\
&- \beta_c p0^f O_1^t O_2^t \\
FaPS_i^{out} &= \gamma S^{free} (p0^f O_1^t O_2^f + 2p0^f O_1^t O_2^t + p0^f O_1^f O_2^t) \\
SiPS^{in} &= -FaPS_i^{out}
\end{aligned}
$$

The YFP Pool

Species and fluxes

$FaPS^{in}$	flux of YFP from the corresponding mRNA Pool
YFP	YFP monomers

Reactions

$$
\begin{aligned}
FaPS^{in} &\Longrightarrow YFP \\
YFP &\longrightarrow \quad k_{dp}
\end{aligned}
$$

Polymerase, ribosome, spliceosome, and IPTG Pools

These Pools do not contain any reaction. They store molecules not bound to the DNA or the mRNA. The RNA Polymerase Pool exchanges a balance flux with each promoter in the nucleus and gets an input flux from every terminator; the ribosome Pool exchanges a balance flux and gets an input flux from each mRNA Pool in the cytoplasm; the spliceosome Pool exchanges a balance flux with each coding region, the IPTG Pool exchanges a balance flux with the LacI Pool and sends an output flux to the pLacOp2 promoter. Since a balance flux is the sum of an input and an output flux, the dynamics of the free molecules stored in these Pool is given by the following ordinary differential equation:

$$
\frac{d\,molecules^{free}}{dt} = flux^{in} - flux^{out} = flux^b
$$

AND lacOp-tetOp

The AND lacOp-tetOp gate differs from the YES lacOp2 gate for the presence in the nucleus of a third transcription unit producing TetR and the promoter leading the synthesis of Citrine (pLacOpTetOp). Here, we give the modelling of the only pLacOpTetOp promoter since the models of the other Parts and Pools in the circuit are identical to the ones presented above.

Regulated pLacOpTetOp promoter

Species and fluxes

Pol^{free}	RNA polymerases available in their Pool
R^{a1}	free active LacI available in their Pool
R^{i1}	free inactive LacI
R^{a2}	free active TetR available in their Pool
R^{i2}	free inactive TetR
S^1	IPTG molecules available in their Pool
S^2	tetracycline molecules available in their Pool
$p0^fO_1^fO_2^f$	completely free promoter
$p0^tO_1^fO_2^f$	RNA polymerase bound to the promoter
$p0^fO_1^tO_2^f$	LacI bound to its target operator O_1
$p0^fO_1^fO_2^t$	TetR bound to its target operator O_2
$p0^fO_1^tO_2^t$	LacI bound to O_1 and TetR bound to O_2
Pol^{cl}	RNA polymerase in the promoter cleaning phase
Pol^{cl}_{lk}	RNA polymerase in the promoter cleaning phase due to promoter leakage
$PoPS^b$	exchanged with the polymerase Pool
$PoPS^{out}$	sent to the YFP coding region
$PoPS^{out}_{lk}$	sent to the YFP mRNA Pool in the cytoplasm
$FaPS^{b1}_a$	flux of active LacI exchanged with the LacI Pool
$FaPS^{b2}_a$	flux of active TetR exchanged with the TetR Pool
$FaPS^{out1}_i$	flux of inactive LacI sent to the LacI Pool
$FaPS^{out2}_i$	flux of inactive TetR sent to the TetR Pool
$SiPS^{in1}$	flux of IPTG molecules from their Pool
$SiPS^{in2}$	flux of tetracycline molecules from their Pool

Reactions

$$
\begin{aligned}
Pol^{free} + p0^fO_1^fO_2^f &\longleftrightarrow p0^tO_1^fO_2^f && k_1, k_{-1}\\
p0^tO_1^fO_2^f &\longrightarrow p0^fO_1^fO_2^f + Pol^{cl} && k_2\\
R^{a1} + p0^fO_1^fO_2^f &\longleftrightarrow p0^fO_1^tO_2^f && \alpha_{lac}, \beta_{lac}\\
R^{a1} + p0^fO_1^fO_2^t &\longleftrightarrow p0^fO_1^tO_2^t && \alpha_{lac}, \beta_{lac}\\
R^{a2} + p0^fO_1^fO_2^f &\longleftrightarrow p0^fO_1^fO_2^t && \alpha_{tet}, \beta_{tet}\\
R^{a2} + p0^fO_1^tO_2^f &\longleftrightarrow p0^fO_1^tO_2^t && \alpha_{tet}, \beta_{tet}\\
S^1 + p0^fO_1^tO_2^f &\longrightarrow p0^fO_1^fO_2^f + R^{i1} && \gamma_{lac}\\
S^1 + p0^fO_1^tO_2^t &\longrightarrow p0^fO_1^fO_2^t + R^{i1} && \gamma_{lac}\\
S^2 + p0^fO_1^fO_2^t &\longrightarrow p0^fO_1^fO_2^f + R^{i2} && \gamma_{tet}\\
S^2 + p0^fO_1^tO_2^t &\longrightarrow p0^fO_1^tO_2^f + R^{i2} && \gamma_{tet}\\
p0^fO_1^tO_2^f &\longrightarrow p0^fO_1^fO_2^f && k_{dp}\\
p0^fO_1^tO_2^t &\longrightarrow p0^fO_1^fO_2^t && k_{dp}\\
p0^fO_1^fO_2^t &\longrightarrow p0^fO_1^fO_2^f && k_{dp}\\
p0^fO_1^tO_2^t &\longrightarrow p0^fO_1^tO_2^f && k_{do}
\end{aligned}
$$

Fluxes calculation

$$
\begin{aligned}
PoPS^{out} &= k_2 p0^t O_1^f O_2^f \\
PoPS^{out}_{lk} &= k_2^{lk}(p0^f O_1^f O_2^t + p0^f O_1^t O_2^f + p0^f O_1^t O_2^t) \\
PoPS^b &= k_1 Pol^{free} p0^f O_1^f O_2^f - k_{-1} p0^t O_1^f O_2^f \\
FaPS^{b1}_a &= \alpha_{lac} R^{a1}(p0^f O_1^f O_2^f + p0^f O_1^f O_2^t) + \\
&- \beta_{lac}(p0^f O_1^t O_2^f + p0^f O_1^t O_2^t) \\
FaPS^{b2}_a &= \alpha_{tet} R^{a2}(p0^f O_1^f O_2^f + p0^f O_1^t O_2^f) + \\
&- \beta_{tet}(p0^f O_1^f O_2^t + p0^f O_1^t O_2^t) \\
FaPS^{out1}_i &= \gamma_{lac} S^1(p0^f O_1^t O_2^f + p0^f O_1^t O_2^t) \\
FaPS^{out2}_i &= \gamma_{tet} S^2(p0^f O_1^f O_2^t + p0^f O_1^t O_2^t) \\
SiPS^{in1} &= -FaPS^{out1}_i \\
SiPS^{in2} &= -FaPS^{out2}_i
\end{aligned}
$$

Notice that the model for the pLacOp promoter used in our computational analysis is the same as the one for pLacOpTetOp after removing the O_2 operator.

Bibliography

[1] A. Belle, A. Tanay, L. Bitincka, R. Shamir, and E. K. O'Shea. Quantification of protein half-lives in the budding yeast proteome. *Proc Natl Acad Sci U S A*, 103(35):13004–13009, Aug 2006.

[2] D. Endy, I. Deese, and the MIT Synthetic Biology Working Group. Adventures in Synthetic Biology. Appeared in Foundations for engineering biology p449, *Nature* **438**, 449-453, 2005.

[3] P. Jorgensen, N. P. Edgington, B. L. Schneider, I. Rupes, M. Tyers, and B. Futcher. The size of the nucleus increases as yeast cells grow. *Mol Biol Cell*, 18(9):3523–3532, Sep 2007.

[4] P. Jorgensen, J. L. Nishikawa, B.-J. Breitkreutz, and M. Tyers. Systematic identification of pathways that couple cell growth and division in yeast. *Science*, 297(5580):395–400, Jul 2002.

[5] M. A. Marchisio, M. Colaiacovo, E. Whitehead, and J. Stelling. Modular, rule-based modeling for the design of eukaryotic synthetic gene circuits. *BMC Syst Biol*, 7:42, 2013.

[6] M. A. Marchisio and J. Stelling. Computational design of synthetic gene circuits with composable parts. *Bioinformatics*, 24(17):1903–1910, Sep 2008.

[7] M. A. Marchisio and J. Stelling. Simplified computational design of digital synthetic gene circuits. In K. R. G.-B. Stan, V. Kulkarni, editor, *System Theoretic and Computational Perspectives in Systems and Synthetic Biology (in press)*. Springer-Verlag, 2013.

[8] T. T. Marquez-Lago and M. A. Marchisio. Synthetic biology: Dynamic modeling and construction of cell systems. In M. Georgiadis, J. Banga, and E. Pistikopoulos, editors, *Dynamic Process Modeling*, volume 7 of *Process Systems Engineering.*, pages 493–544. WILEY-VCH, 2010.

[9] S. E. Munchel, R. K. Shultzaberger, N. Takizawa, and K. Weis. Dynamic profiling of mrna turnover reveals gene-specific and system-wide regulation of mrna decay. *Mol Biol Cell*, 22(15):2787–2795, Aug 2011.
